# Antagonistic and Synergistic Activation of Cardiovascular Vagal and Sympathetic Motor Outflows in Trigeminal Reflexes

**DOI:** 10.3389/fneur.2017.00052

**Published:** 2017-02-21

**Authors:** Bruno Buchholz, Jazmín Kelly, Eduardo A. Bernatene, Nahuel Méndez Diodati, Ricardo J. Gelpi

**Affiliations:** ^1^Facultad de Medicina, Departamento de Patología, Instituto de Fisiopatología Cardiovascular (INFICA), Universidad de Buenos Aires, Buenos Aires, Argentina; ^2^Facultad de Medicina, Consejo Nacional de Investigaciones Científicas y Técnicas (CONICET), Instituto de Bioquímica y Medicina Molecular (IBIMOL), Universidad de Buenos Aires, Buenos Aires, Argentina; ^3^Consejo Nacional de Investigaciones Científicas y Técnicas (CONICET), Buenos Aires, Argentina

**Keywords:** trigeminocardiac reflex, diving reflex, heart, arrhythmia, myocardial ischemia

## Abstract

The trigeminal nerve and heart are strongly related through somato-autonomic nervous reflexes that induce rapid changes in cardiovascular function. Several trigeminal reflexes have been described, but the diving and trigeminocardiac reflexes are the most studied. The heart is a target organ dually innervated by the sympathetic and parasympathetic systems. Thus, how cardiac function is regulated during the trigeminal reflexes is the result of the combination of an increased parasympathetic response and increased, decreased, or unaltered sympathetic activity. Various hemodynamic changes occur as a consequence of these alterations in autonomic tone. Often in the oxygen-conserving physiological reflexes such as the diving reflex, sympathetic/parasympathetic co-activation reduces the heart rate and either maintains or increases blood pressure. Conversely, in the trigeminocardiac reflex, bradycardia and hypotension due to parasympathetic activation and sympathetic inactivation tend to be observed. These sudden cardiac innervation disturbances may promote the generation of arrhythmias or myocardial ischemia during surgeries in the trigeminal territory. However, the function and mechanisms involved in the trigeminal reflexes remain to be fully elucidated. The current review provides a brief update and analysis of the features of these reflexes, with special focus on how the autonomic nervous system interacts with cardiovascular function.

## Introduction

Physiological or pathological stimulation of the trigeminal nerve can trigger sudden cardiovascular disturbances with the characteristic features of a nervous reflex (1). Although these trigeminal reflexes have been thoroughly described in numerous clinical–surgical situations, their physiological and pathophysiological mechanisms, as well as their functional significance, have not been elucidated. In 1908, Aschner (2) and Dagnini (3) described a severe reduction in heart rate as a consequence of eyeball compression (4). In 1975, Kumada et al. described the trigeminal depressor response in an experimental animal model (5). They observed that stimulation of one of the trigeminal branches or its nuclear sensitive complex triggered a reflex that induced cardiovascular symptoms including a sharp reduction of heart rate, hypotension, apnea, and gastric hypermotility (6, 7). In 1988, Shelly and Church suggested the term “trigeminocardiac reflex” (8), and in 1991, Lang et al. used the term trigeminocardiac reflex to describe intense reflex bradycardia observed in three patients undergoing maxillofacial surgeries (9). In later years, Schaller and colleagues (10, 11) first published the occurrence of a central reflex in humans during cerebellopontine angle and brainstem surgeries and merged these peripheral and central responses into a single autonomic reflex, which is now generally accepted as the trigeminocardiac reflex.

These reflexes represent somato-autonomic responses where the trigeminal nerve is the afferent pathway, the vagus and sympathetic nerves are the efferent pathways, and numerous brainstem nuclei serve as integration centers (12). Although these reflexes share certain anatomical structures, the stimuli by which they are triggered and the responses they elicit are not necessarily equal. The trigeminal reflexes can be triggered by stimuli sensed by thermoreceptors in the facial skin (diving reflex) (13), nasal mucosa (nasopharyngeal reflex) (14), and eyeball (oculocardiac reflex) (4, 15). They can also be activated by direct stimulation of some trigeminal branches and the trigeminal nuclear complex of the brainstem (trigeminocardiac reflex) (16).

## Trigeminocardiac Reflex

The trigeminocardiac reflex is a brainstem reflex that has been demonstrated both clinically and experimentally (17). Schaller et al. defined the reflex from a clinical point of view as hypotension with a 20% drop in mean arterial blood pressure and bradycardia lower than 60 beats/min in response to surgical manipulation of the trigeminal nerve trunk or disturbances in the territory of one of its branches. This concept was later redefined by including autonomic symptoms such as a decrease in cardiovascular function less than 20%. This new trigeminocardiac reflex definition is even more inclusive for clinical studies (12). In severe instances, this response can sometimes lead to asystole. They can be classified into two subtypes, depending on which sensory territory is stimulated: the central trigeminocardiac reflex (ganglion to nucleus) and the peripheral trigeminocardiac reflex (peripheral divisions to ganglion). The peripheral trigeminocardiac reflex can be further subdivided into ophthalmocardiac and maxillomandibulocardiac reflexes (16).

**Figure 1:**
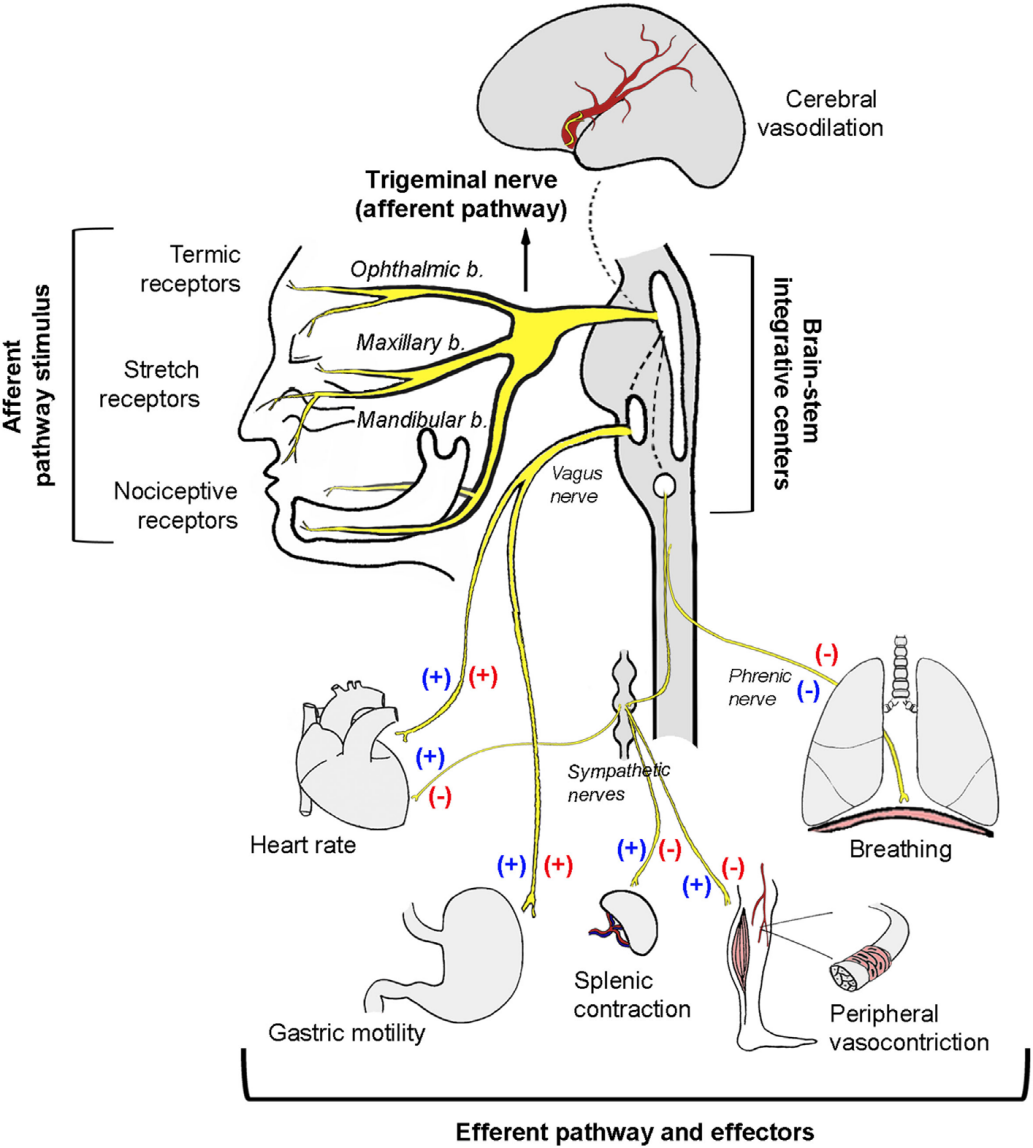
**Schematic illustration of the autonomic neural pathways and effectors activated as a consequence of trigeminal nerve stimulation**. Protection reflexes like the diving, the nasopharyngeal, or the oculocardiac reflex involve simultaneous co-activation of both autonomic limbs (blue symbols). The trigeminocardiac reflex induces a strong depressor response by a reciprocal activation of the parasympathetic system and an inhibition of the sympathetic system (red symbols).

In anesthetized rabbits, trigeminal system stimulation reduced heart rate by 13%, mean arterial pressure by 36%, total peripheral vascular resistance by 35%, and cardiac output by a comparatively modest 5%, whereas stroke volume increased by 6% (7). Bilateral vagal section does not reverse hypotension and only partially reverses the decrease in heart rate. This experiment demonstrates that hypotension is independent of heart rate reduction and occurs as a result of vasodilation induced by the sustained decrease in peripheral vascular sympathetic tone. In contrast, heart rate reduction occurs *via* a combination of parasympathetic activation and sympathetic inhibition. Using a different experimental model in rabbits breathing spontaneously, Kumada et al. (6) studied the respiratory effects of electrical stimulation of the spinal trigeminal tract and its nuclei. In this research, a biphasic response could be observed: low frequency and low intensity stimulation produced tachypnea, whereas a slightly more intense stimulation led to expiratory apnea. On the other hand, gastric hypermotility as a consequence of increased vagal tone was also observed, thus constituting itself as an actual trigeminovagal reflex (6) (Figure 1). Based on hemodynamic analysis, the trigeminocardiac reflex behaves in a similar manner to the baroreceptor reflexes, in which bradycardia and reduced systemic blood pressure can also be observed as a consequence of vagal activation and sympathetic inhibition (7). As a variant of the trigeminocardiac reflex, the oculocardiac reflex generates the same synergistic sympathetic/parasympathetic output and produces bradycardia and hypotension (Table 1).

**Table 1:** **Summary of the principal characteristics of the trigeminal reflex subtypes**.

Trigeminal reflex	Triggered by	Efferent pathway	Arterial pressure	Heart rate	Gastric motility	Splenic contraction	Peripheral vascular tone	Breathing
Trigeminocardiac reflex	Direct stimulation of the trigeminal nerve	Parasympathetic ↑	↓	↓	↑	?	↓	Apnea
		Sympathetic ↓						
Diving reflex	Thermoreceptors in the facial skin	Parasympathetic ↑	↑	↓	–	↑	↑	Apnea
		Sympathetic ↑						
Nasopharyngeal reflex	Nasal mucosa irritation	Parasympathetic ↑	=	↓	–	↑	↑	Apnea
		Sympathetic ↑						
Oculocardiac reflex	Physical stimulation of the eye or adnexa	Parasympathetic ↑	↓	↓	↑	?	↓	Apnea
		Sympathetic ↑						

*?, Not known*.

## Diving Reflex

Oxygen deprivation, even for brief periods of time, can be highly detrimental. However, many species, such as diving birds, mammals, and even human beings, have adapted to withstand hypoxia or anoxia for longer periods (18). One of the most important physiological adaptations that allow these animals to withstand the lack of oxygen during apnea is the diving reflex (13). Facial submersion in water rapidly triggers a heart rate reduction by vagal activation, an increase in blood pressure by sympathetic hyperactivity, and apnea. In humans, the autonomic response can often be intense: the heart rate can drop to 20–30 beats/min, and the increased peripheral vascular resistance can raise blood pressure to critical levels. This is not observed in other species that are specialized in the art of diving because they are able to maintain blood pressure within physiological ranges despite increased sympathetic tone.

An important modulator of autonomic activity during diving is apnea, which can result by two different mechanisms. First, apnea can occur voluntarily; in this situation, a person consciously inhibits the respiratory centers *via* a centrally induced pathway. An example is simple breath holding. Second, apnea can occur in a reflexive manner following stimulation of cold receptors in the facial skin, eyes, and nasal cavity. At the same time, changes in pulmonary volumes due to apnea can modify the autonomic tone, leading to cardiovascular changes (13). Apnea alone is sufficient to trigger the diving response; however, a greater response is seen when coupled with stimulation of facial cold receptors, as with face immersion. This particularly intense cardiovascular reflex response manifests itself as a consequence of both facial stimulation and apnea. Fagius and Sundlöf studied sympathetic activity in the peroneal nerve and skin of patients after cold water submersion and reported differences in autonomic regulation between muscle and cutaneous blood flow. They observed increased activity of the nerves innervating muscle vessels, accompanied by 30–50% reduction in blood flow as a consequence of higher peripheral vascular resistance (19). However, they also found decreased sympathetic conductivity toward the cutaneous vascular beds, which have a larger role in thermoregulation and sweating. Increased sympathetic tone in the splanchnic vessels and splenic capsule was also observed (18). The contraction of the spleen provides an additional blood volume that aids in stabilizing blood pressure despite the decreased heart rate. Since contraction of the spleen also increases hematocrit and hemoglobin circulating in the blood, it would be expected that oxygen transport would also be enhanced.

The diving reflex in human beings can be modified by many factors, but the most important are water temperature, oxygen tension in the arterial blood, and emotional factors (20). Previous studies showed a clear inverse relationship between the temperature of the water in which the face is immersed and the magnitude of the diving bradycardia. Interestingly, there may not be a similar close dependence on temperature for the reduction in limb blood flow. With respect to blood oxygen, diving bradycardia is increased if subjects have been breath holding or exercising immediately before performing apnea with face immersion. Such observations suggest the possibility that a reduction in the arterial oxygen tension potentiates the diving reflex. Finally, higher brain functions have profound effects on the development of the diving reflex in humans. When subjects are distracted, diving bradycardia fails to occur despite the face being under water. On the other hand, fear can powerfully accentuate the diving response.

Physiologically, the diving reflex slows down oxygen uptake from the lungs and reduces the rate of arterial blood desaturation, slowing the depletion of both lung and blood oxygen stores. In addition, blood flow is redistributed so that the brain and heart are preferentially perfused. This reduces oxygen delivery to the peripheral capillary beds by stopping blood flow with intense vasoconstriction (18). Therefore, the diving reflex is one of the most powerful somato-autonomic reflexes of the organism, and it is an important life protection mechanism in naturally diving animals. As one might expect, the usual response in the human being, a terrestrial animal, is quantitatively less efficient than that seen in the natural divers.

## Nasopharyngeal Reflex

This is a well-demonstrated variation of the diving reflex, activated by stimulation of the nasal mucosa (14). The nasopharyngeal reflex can be activated by irritating gases, water, or electrical stimulation. The ethmoidal nerve plays an important role in this mechanism by innervating the nasal passages and external nares, and it is fundamental in protecting the upper airway (21, 22). Studies in dogs verified that nasal stimulation increases parasympathetic vagal tone, which reduces heart rate and, consequently, cardiac output. Conversely, the nasopharyngeal reflex increases sympathetic tone, leading to greater peripheral vascular resistance (with the exception of carotid resistance), therefore maintaining stable blood pressure values (23) (Table 1). The differential changes in vascular flow at the carotid level suggest blood flow redistribution to the brain. The aforementioned physiological features of the nasopharyngeal reflex demonstrate its role as a powerful oxygen-preserving reflex, similar to the diving reflex.

## Antagonism and Synergism in Sympathetic/Parasympathetic Interaction

Although the trigeminal reflexes have many aspects in common (e.g., the anatomical substrate), their cardiovascular responses may differ depending on the sympathetic/parasympathetic interaction (24). As already mentioned, the diving reflex elicits strong synergistic co-activation of the sympathetic/parasympathetic systems, allowing for heart rate reduction with concurrent maintenance or increase of peripheral arterial pressure (13, 14). On the other hand, the trigeminocardiac reflex is characterized by parasympathetic activation and sympathetic inhibition, which simultaneously reduce heart rate and blood pressure (6) (Figure 1; Table 1).

Some studies have clarified the factors that condition trigeminal stimulation responses. First, the type of stimulus triggering the reflex should be taken into consideration. Pressor responses are frequently reported, indicating an increase in the peripheral sympathetic tone in response to physiological stimuli, such as irritation of the nasal mucous membrane (14) or stimulation of the thermal nerve endings of facial skin (25). Conversely, in surgical or experimental stimulations of the trigeminal nerve trunk in anesthetized patients, important reductions can be observed both in sympathetic tone and blood pressure (16). Stimulus intensity, frequency, and duration, as well as the type of afferent nervous fibers involved in the reflexogenic response, should also be taken into account.

The second aspect to consider is the administration of different analgesics and anesthetics that may modulate the trigeminal–vagal reflexes in variable and unpredictable ways (26–29). This is of particular importance for the trigeminocardiac reflex, since numerous studies reported autonomic reaction differences based on the use of these drugs (30). Therefore, anesthesia type and depth are important factors to consider in the clinical management of patients undergoing surgical interventions in the trigeminal territory who are at potential risk of cardiovascular depression (31–33).

The last aspect to consider is species differences. In diving animals such as seals, the diving reflex increases sympathetic tone and allows preservation of blood pressure within physiological ranges. In humans, however, the sympathetic response of the diving reflex is more intense and may increase blood pressure to critical values (30).

## Hemodynamic Changes During Trigeminal Reflexes

Bradycardia and blood pressure modifications are the most evident hemodynamic changes induced by these reflexes. The sudden changes that occur in these reflexogenic adaptations may impact patients with cardiovascular disease, inducing severe and sometimes lethal complications. Various authors reported complications in patients with coronary disease, and severe arrhythmias such as asystole and ventricular fibrillations as a consequence of the trigeminocardiac reflex during neurosurgeries (34, 35). Although not experimentally demonstrated, coronary spasm might be a key factor in the pathophysiology of these complications. Cholinergic discharge in coronary arteries damaged by atherosclerosis could trigger paradoxical vasoconstriction. Also, the sympathetic co-activations that often occur due to trigeminal reflexes may produce vasospasm and decreased blood flow to the myocardium. This cardiac sympathetic co-activation was studied by Nalivaiko et al. in rabbits *via* electrocardiogram, which confirmed the presence of profound co-activation during the nasopharyngeal reflex (36). Intense changes in autonomic tone following trigeminal stimulation may engender electrical instabilities, which in turn may predispose patients to arrhythmias and imbalances between myocardial oxygen supply and demand.

We previously demonstrated that short-term vagal electro-stimulation in rabbits can generate critical changes in myocardial oxygen consumption in the context of coronary ischemia and reperfusion (37). It is interesting to consider that oxygen consumption may vary intensely, to the point of increasing or reducing infarct size depending on the sympathetic/parasympathetic interaction (38). When vagal activation antagonizes the sympathetic system, both infarct size and oxygen consumption are reduced. However, we observe an opposite situation when the sympathetic system is co-activated during vagus nerve stimulation. This shows how both divisions of the autonomic nervous system interaction is fundamental for correct interpretation of the trigeminal reflexes. Even though by definition a major vagal discharge is present, the sympathetic tone fluctuates between an increase and a decrease. It is therefore a critical variable to consider because it can develop numerous complications that may be catastrophic during medical interventions in the trigeminal nerve territory.

## Conclusion

Stimulation of the trigeminal nerve or the territories innervated by its branches can trigger deep hemodynamic changes due to neurogenic somato-autonomic responses such as the diving and trigeminocardiac reflexes. Although they can act as extremely efficient oxygen-conserving reflexes, the sudden disturbances of cardiovascular autonomic tone can often generate electrical instabilities, predisposing to cardiac arrhythmias and myocardial ischemia. Differences in hemodynamic responses to trigeminal stimulation depend upon the antagonistic or synergistic interaction of the sympathetic and parasympathetic systems. Future investigations will be needed to understand the molecular mechanisms and functional purposes of the trigeminal reflexes. This knowledge is of paramount importance for appropriate management of patients affected during surgeries in the trigeminal territory.

## Author Contributions

BB brought the manuscript to fruition through the collection of historical and current trends in trigeminal reflexes and composed early drafts of the manuscript and figure. JK contributed to each section including major revisions, current trends, and research in each aspects of the manuscript. EB and ND contributed to revisions and facilitated with manuscript editing and figure assembly. RG provided critical feedback and manuscript revisions, significant intellectual commentary on the manuscript, and final approval of the version to be published.

## Conflict of Interest Statement

The authors declare that the research was conducted in the absence of any commercial or financial relationships that could be construed as a potential conflict of interest.
